# Associations of Dietary Vitamin C and E Intake With Depression. A Meta-Analysis of Observational Studies

**DOI:** 10.3389/fnut.2022.857823

**Published:** 2022-04-07

**Authors:** Jun Ding, Yi Zhang

**Affiliations:** ^1^Changsha Social Work College, Changsha, China; ^2^Department of Orthopaedics, Xiangya Hospital, Central South University, Changsha, China; ^3^National Clinical Research Center for Geriatric Disorders, Xiangya Hospital, Central South University, Changsha, China

**Keywords:** dietary vitamin C, dietary vitamin E, depression, meta-analysis, observational studies

## Abstract

**Objective:**

The associations of dietary vitamin C and E intake with depression remains conflicting. This meta-analysis of observational study was therefore employed to clarify the issue further.

**Methods:**

An extensive literature review (PubMed, Web of Science and Embase) was performed in January 2022 to identify the observational studies on the associations of dietary vitamin C and E intake with depression. The pooled relative risk (RR) of depression for the highest versus lowest dietary vitamin C and E intake category, and the weighted mean difference (WMD) of dietary vitamin C and E intake for depression versus control subjects, were calculated.

**Results:**

A total of 25 observational studies (91966 participants) were included in this meta-analysis. The overall multi-variable adjusted RR demonstrated that dietary vitamin C intake was inversely associated with depression (RR = 0.72, 95% CI: 0.57 to 0.91; *P* = 0.005). In addition, the combined WMD showed that the dietary vitamin C intake in depression was lower than that in control subjects (WMD = −11.58, 95% CI: −14.88 to −8.29; *P* < 0.001). Similarly, the overall multi-variable adjusted RR demonstrated that dietary vitamin E intake was negatively associated with depression (RR = 0.84, 95% CI: 0.72 to 0.98; *P* = 0.02). Moreover, the combined WMD showed that the dietary vitamin E intake in depression was also lower than that in control subjects (WMD = −0.71, 95% CI: −1.07 to −0.34; *P* < 0.001).

**Conclusion:**

The results of this meta-analysis suggest that both dietary vitamin C and E intake is inversely associated with depression. However, due to the limited evidence, more well-designed prospective cohort studies are still needed.

## Introduction

As one of the most common mental disorders, depression affects females twice as much as males worldwide (1). The symptoms of depression are usually presented as exhaustion, sadness, lack of interest in daily activities, and even suicide (2). Affecting approximately 300 million people (3), depression is estimated to be the leading cause of disability worldwide by 2030 (4). Nevertheless, the treatment for depression is usually restricted to unsatisfactory curative effect, adverse side effects and costly pharmacotherapy (5). Recently, epidemiological evidence indicates that the dietary factors are associated with depression (6, 7). Thus, the identification of modifiable dietary factors for depression appears to be an important step in its clinical management.

Vitamin C (ascorbic acid), an essential water-soluble micronutrient, is traditionally employed to prevent and treat scurvy (8). However, vitamin E (also known as tocopherol) is a fat-soluble vitamin to modulate enzymes involved in signal transduction, gene expression and immunomodulatory capabilities (9, 10). Equipped with abundant vitamin C and E constituent, vegetable, fruit, legume and nut consumption are demonstrated to be inversely associated with depression (11–13). It is well known that vitamin C and E are served as common antioxidants that prevent other compounds from being oxidized (14–16), which donate electron and scavenge harmful free radicals. Since the oxidative stress is also considered to play a significant role in the pathophysiology of depression (17, 18), it seems naturally that dietary vitamin C and E intake is negatively associated with depression.

As far as we know, a number of observational studies have investigated the associations of dietary vitamin C and E intake with depression (19–43). However, their results are still conflicting. Therefore, this meta-analysis is employed to clarify the issue further. It is hypothesized that both dietary vitamin C and E intake is inversely associated with depression.

## Materials and Methods

### Search Strategy

We performed our meta-analysis according to the Preferred Reporting Items for Systematic Reviews and Meta-analyses (PRISMA) guidelines (44). The PubMed, Web of Science and Embase electronic database were searched during January 2022 by using a combination of keywords and in-text words related to depression (‘depression,’ ‘depressive’), vitamin C (‘vitamin C,’ ‘ascorbic acid’) and vitamin E (‘vitamin E,’ ‘tocopherol’). No language restrictions were imposed. The titles and abstracts of all articles were first screened to identify eligible studies, and then the full articles were read to include the eligible studies. Moreover, the reference lists for the retrieved articles were also reviewed to identify additional studies.

### Study Selection

Two researchers reviewed the titles, abstracts and full texts of all retrieved studies independently. Disagreements, if any, were resolved by discussions. The included studies were required to meet the following criteria: (1) observational studies; (2) the associations of dietary vitamin C and E intake with depression; and (3) relative risk (RR), odds ratio (OR) or weighted mean difference (WMD) with 95% confidence interval (CI) reported. The exclusion criteria were listed as follows: (1) duplicated or irrelevant articles; (2) reviews, letters or case reports; (3) randomized controlled trials; and (4) non-human studies.

### Data Extraction

Two researchers extracted the data independently, and disagreements were resolved by discussion. The information about first author, year of publication, location, age, sex, sample size, study design, adjustments, dietary assessment, category of exposure, effect estimates and diagnostic criteria of depression, was collected respectively. The corresponding effect estimates with 95% CIs for the highest versus lowest dietary vitamin C and E intake category were extracted (adjusted for the maximum number of confounding variables). Moreover, the dietary vitamin C and E intake (mean ± SD) was also extracted for depression versus control subjects to calculate the WMD by Review Manager 5.3. The quality assessment was employed in accordance with the Newcastle-Ottawa Scale (NOS), which contains 8 items categorized into three dimensions: the selection of study groups, the comparability among different groups, and the ascertainment of either the exposure or outcome for case-control (Supplementary Table 1) and cohort studies (Supplementary Table 2), respectively.

### Statistical Analyses

The RR for depression and WMD for dietary vitamin C and E intake were the outcome measures in our study. The I^2^ statistic, which measures the percentage of total variation across studies due to heterogeneity, was examined (*I*^2^ > 50% was considered heterogeneity). If significant heterogeneity was observed among the studies, the random-effects model was used; otherwise, the fixed effects model was utilized. Univariate meta-regression for publication year, sample size, location, age, sex, and dietary assessment was performed to explore the sources of heterogeneity. Begg’s test was employed to assess the publication bias (45, 46). Moreover, the subgroup analysis was employed for geographical region, dietary assessment, sex, population, study design, and diagnostic criteria of depression, respectively. In addition, a sensitivity analysis was also conducted to determine whether an individual study affected the pooled result.

## Results

### Study Identification and Selection

**FIGURE 1:**
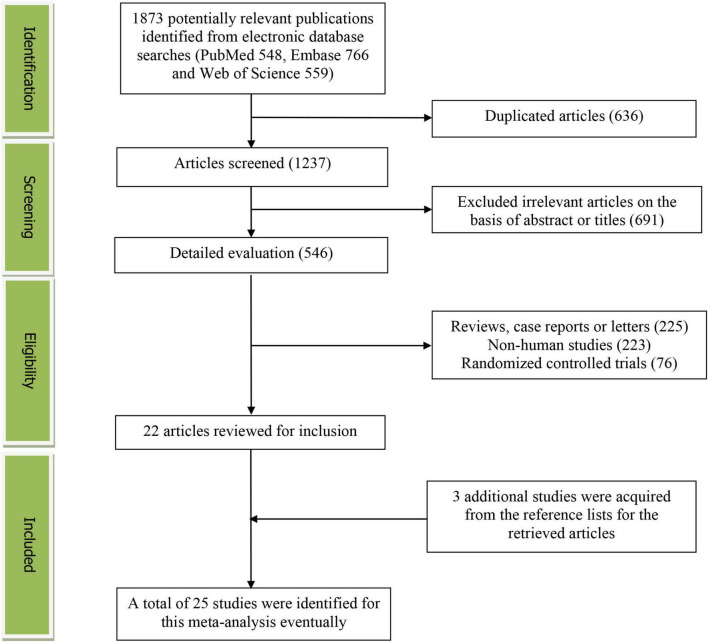
The detailed flow diagram of the study identification and selection in this meta-analysis.

The detailed flow diagram of the study identification and selection was presented in Figure 1. A total of 1873 potentially relevant articles (548 for PubMed, 766 for Embase and 559 for Web of Science) were retrieved during the initial literature search. After eliminating 636 duplicated articles, 1237 articles were screened according to the titles and abstracts. 691 irrelevant studies were excluded. Then, 225 reviews, case reports or letters, 223 non-human studies, 76 randomized control trials studies were removed. Thereafter, 3 additional studies were acquired from the reference lists for the retrieved articles. Eventually, 25 studies were selected for this meta-analysis (19–43).

### Study Characteristics

The main characteristics of the included studies were presented in Table 1. These studies were published between 2009 and 2022. 12 of the included studies were performed in Asian countries [Korea (21, 26, 28, 34, 35, 41), Iran (25, 37, 39) and Japan (19, 20, 31)], and the other ones were conducted in United States (22, 24, 33, 40, 43), Brazil (32, 38, 42), Australia (23, 36), Spain (29, 30), and Turkey (27). Male, female and both male and female participants were recruited in 2 (25, 36), 8 (21, 26, 32, 34, 37–39, 43), and 15 (19, 20, 22–24, 27–31, 33, 35, 40–42) studies, respectively. The sample size ranged from 41 to 25895 for a total number of 91966. The dietary vitamin C and E intake was assessed by food-frequency questionnaire (FFQ) in 14 studies (19, 20, 22, 23, 25, 26, 28, 30, 31, 36, 37, 39, 42, 43), and recall method in 12 studies (21, 24, 25, 27, 29, 32–35, 38, 40, 41). The diagnostic criteria of depression or depressive symptom were Diagnostic and Statistical Manual of Mental Disorders-IV (DSM-IV) (27, 30), Patient Health Questionnaire-9 (PHQ-9) (24, 33, 35, 40), Center for Epidemiological Studies Depression Scale (CES-D) (19–21, 23, 29, 34, 38, 43), Beck Depression Inventory (BDI) (25–27, 32, 39), Geriatric Depression Scale (GDS) (31, 36), Depression, Anxiety, Stress Scale (DASS) (37), National Institute of Mental Health (NIMH) (22), and Clinical Interview Schedule Revised (CIS-R) (42), respectively.

**TABLE 1:** Characteristics of the individual studies included in this meta-analysis.

First author year of publication	Location	Age years	Sex	Sample size	Study design	Adjustments	Dietary assessment	Category of exposure	Effect estimates (RR or WMD)	Diagnostic criteria of depression	NOS
Oishi 2009 (19)	Japan	65-75	Both	279	Cross-sectional	Age, chronic diseases, BMI and social support	FFQ	Male Vitamin C Tertile 1 Tertile 2 Tertile 3 Vitamin E Tertile 1 Tertile 2 Tertile 3 Female Vitamin C Tertile 1 Tertile 2 Tertile 3 Vitamin E Tertile 1 Tertile 2 Tertile 3	1.00 0.55 (0.21, 1.47) 0.33 (0.12, 0.93) 1.00 0.33 (0.12, 0.92) 0.39 (0.14, 1.08) 1.00 0.55 (0.22, 1.43) 0.47 (0.18, 1.22) 1.00 0.70 (0.27, 1.83) 0.76 (0.29, 1.98)	CES-D	8
Nanri 2010 (20)	Japan	21-67	Both	521	Cross-sectional	NA	FFQ	Control Depression Control Depression	Vitamin C (mg/day) 63.6 (60.9, 66.3) 60.5 (56.5, 64.5) Vitamin E (mg/day) 4.2 (4.1, 4.3) 4.0 (3.8, 4.2)	CES-D	7
Park 2010 (21)	Korea	20.5	Female	130	Case-control	NA	Recall method	Control Depression Control Depression	Vitamin C (mg/day) 66.2 (56.4, 76.0) 52.4 (46.0, 58.8) Vitamin E (mg/day) 11.9 (10.9, 12.9) 11.2 (10.2, 11.2)	CES-D	7
Payne 2012 (22)	US	> 60	Both	278	Case-control	NA	FFQ	Control Depression Control Depression	Vitamin C (mg/day) 148.3 (137.1, 159.5) 109.2 (99.0, 119.4) Vitamin E (mg/day) 10.2 (9.3, 11.1) 9.9 (9.0, 10.8)	NIMH	7
Purnomo 2012 (23)	Australia	> 18	Both	58	Case-control	NA	FFQ	Control Depression	Vitamin C (mg/day) 147.5 (120.4, 174.6) 142.8 (101.3, 184.3)	CES-D	5
Beydoun 2013 (24)	US	20-85	Both	1798	Cross-sectional	NA	Recall method	Male Control Depression Control Depression Female Control Depression Control Depression	Vitamin C (mg/day) 91.6 (83.7, 99.5) 101.4 (74.1, 128.7) Vitamin E (mg/day) 8.6 (8.0, 9.2) 6.9 (5.7, 8.1) Vitamin C (mg/day) 82.7 (77.6, 87.8) 67.1 (52.1, 82.1) Vitamin E (mg/day) 6.9 (6.7, 7.1) 5.5 (4.7, 6.3)	PHQ-9	8
Prohan 2014 (25)	Iran	18-25	Male	60	Case-control	NA	FFQ and recall method	Control Depression Control Depression	Vitamin C (mg/day) 106.9 (101.2, 112.6) 94.9 (88.7, 101.1) Vitamin E (mg/day) 11.3 (10.0, 12.6) 11.4 (9.7, 13.1)	BDI	6
Kim 2015 (26)	Korea	15.0	Female	849	Case-control	Menstrual regularity and energy	FFQ	Vitamin C Tertile 1 Tertile 2 Tertile 3 Vitamin E Tertile 1 Tertile 2 Tertile 3	1.00 0.78 (0.47, 1.29) 0.50 (0.27, 0.93) 1.00 0.70 (0.41, 1.20) 0.41 (0.19, 0.87)	BDI	7
Kaner 2015 (27)	Turkey	18-60	Both	59	Case-control	NA	Recall method	Control Depression Control Depression	Vitamin C (mg/day) 97.1 (40.3, 191.9) 45.1 (21.6, 70.3) Vitamin E (mg/day) 9.2 (7.0, 15.4) 7.8 (5.6, 11.7)	DSM-IV	6
Jeong 2016 (28)	Korea	20-65	Both	734	Cross-sectional	NA	FFQ	Male Control Depression Female Control Depression	Vitamin C (mg/day) 116.3 (96.5, 136.1) 98.6 (73.3, 123.9) Vitamin C (mg/day) 116.1 (107.1, 125.1) 122.9 (97.6, 148.2)	BDI	7
Rubio-López 2016 (29)	Spain	6-9	Both	710	Cross-sectional	NA	Recall method	Control Depression Control Depression	Vitamin C (mg/day) 106.0 (102.8, 109.2) 99.1 (93.3, 104.9) Vitamin E (mg/day) 8.1 (7.8, 8.4) 7.3 (6.8, 7.8)	CES-D	7
Villegas 2017 (30)	Spain	38	Both	13983	Cohort	Sex, age, physical activity, BMI, energy intake, special diets, smoking, alcohol intake and prevalence of CVD, HTA or T2DM	FFQ	Vitamin E Inadequacy Adequacy	1.00 0.92 (0.73, 1.15)	DSM-IV	9
Nguyen 2017 (31)	Japan	> 65	Both	1634	Cross-sectional	NA	FFQ	Control Depression Control Depression	Vitamin C (mg/day) 65.8 (63.9, 67.6) 58.6 (55.7, 61.5) Vitamin E (mg/day) 4.0 (3.9, 4.1) 3.1 (3.0, 3.2)	GDS	7
de Oliveira 2019 (32)	Brazil	50-69	Female	41	Case-control	NA	Recall method	Control Depression Control Depression	Vitamin C (mg/day) 127.2 (92.9, 161.4) 98.8 (41.2, 156.5) Vitamin E (mg/day) 4.4 (3.2, 5.7) 5.8 (4.2, 7.4)	BDI	5
Iranpour 2019 (33)	US	> 18	Both	4737	Cross-sectional	NA	Recall method	Control Depression	Vitamin C (mg/day) 87.4 (83.9, 90.9) 71.4 (55.6, 87.2)	PHQ-9	8
Park 2019 (34)	Korea	22	Female	178	Cross-sectional	NA	Recall method	Control Depression	Vitamin C (mg/day) 49.8 (43.7, 55.9) 43.4 (34.0, 52.8)	CES-D	7
Park 2019-2 (35)	Korea	20-60	Both	3381	Cross-sectional	NA	Recall method	Male Control Depression Female Control Depression	Vitamin C (mg/day) 104.4 (99.4, 109.5) 93.0 (84.1 101.8) Vitamin C (mg/day) 116.1 (110.6, 121.6) 95.9 (87.9, 104.0)	PHQ-9	7
Das 2020 (36)	Australia	> 65	Male	794	Cohort	Age, BMI, marital status, living arrangement, income, meal service, smoking, alcohol intake, SRH, PASE, comorbidity, energy, antidepressant medication	FFQ	Vitamin E Quartile 1 Quartile 2 Quartile 3 Quartile 4	1.00 0.35 (0.16, 0.77) 0.50 (0.24, 1.02) 0.49 (0.24, 1.00)	GDS	7
Farhadnejad 2020 (37)	Iran	15-18	Female	263	Cross-sectional	Age, BMI, physical activity, mother/father’s education level, dietary fiber, and total energy intake.	FFQ	Control Depression Control Depression Vitamin C Tertile 1 Tertile 2 Tertile 3 Vitamin E Tertile 1 Tertile 2 Tertile 3	Vitamin C (mg/day) 244.8 (223.2, 266.3) 240.3 (216.9, 263.7) Vitamin E (mg/day) 13.0 (12.2, 13.8) 12.6 (11.7, 13.4) 1.00 0.80 (0.41, 1.55) 0.83 (0.39, 1.75) 1.00 0.74 (0.37, 1.48) 0.51 (0.20, 1.28)	DASS	7
Oldra 2020 (38)	Brazil	40-65	Female	400	Cross-sectional	NA	Recall method	Control Depression	Vitamin C (mg/day) 56.5 (49.8, 63.5) 42.2 (36.2, 48.1)	CES-D	7
Khayyatzadeh 2021 (39)	Iran	12-18	Female	988	Cross-sectional	Age, energy intake, menstruation, family members, parental death, parental divorce, physical activity and BMI	FFQ	Control Depression Control Depression Vitamin C Quartile 1 Quartile 2 Quartile 3 Quartile 4	Vitamin C (mg/day) 99.1 (94.3, 103.9) 90.0 (83.4, 96.6) Vitamin E (mg/day) 13.7 (13.2, 14.2) 13.5 (12.6, 14.4) 1.00 0.81 (0.52, 1.26) 0.68 (0.42, 1.09) 0.61 (0.37, 1.01)	BDI	7
Wang 2021 (40)	US	> 18	Both	25895	Cross-sectional	Age, sex, race, educational level, marital status, body mass index, work physical activity, recreational physical activity, ratio of family income to poverty, smoking status, alcohol consumption, energy, hypertension, diabetes and stroke	Recall method	Control Depression Vitamin C Tertile 1 Tertile 2 Tertile 3	Vitamin C (mg/day) 64.4 (63.3, 65.5) 47.1 (44.0, 50.2) 1.00 0.69 (0.58, 0.83) 0.73 (0.58, 0.91)	PHQ-9	8
Nguyen 2021 (41)	Korea	> 10	Both	16371	Cross-sectional	NA	Recall method	Control Depression	Vitamin C (mg/day) 64.4 (63.3, 65.5) 47.1 (44.0, 50.2)	NA	8
Ferriani 2022 (42)	Brazil	35-74	Both	14737	Cross-sectional	Total calorie, age, race, total cholesterol, HDL cholesterol, systolic blood pressure, antihypertensive drug, diabetes, and smoking, cardiovascular disease and physical activity	FFQ	Control Depression Control Depression Vitamin C Quintile 1 Quintile 2 Quintile 3 Quintile 4 Quintile 5 Vitamin E Quintile 1 Quintile 2 Quintile 3 Quintile 4 Quintile 5	Vitamin C (mg/day) 196.3 (131.9, 282.5) 200.2 (129.6, 295.7) Vitamin E (mg/day) 9.0 (7.4, 11.5) 8.8 (7.3, 11.4) 1.00 0.86 (0.66, 1.11) 0.89 (0.69, 1.15) 0.97 (0.75, 1.24) 1.13 (0.88, 1.45) 1.00 0.85 (0.66, 1.10) 0.90 (0.70, 1.16) 0.80 (0.62, 1.04) 0.94 (0.73, 1.21)	CIS-R	9
Li 2022 (43)	US	42-52	Female	3088	Cross-sectional	age, race/ethnicity, education, financial strain, physical activity, BMI, VMS, use of antidepressant, total caloric intake, n-3 poly- unsaturated fatty acids intake, calcium intake, phosphorus intake, menopausal status, SHBG, testosterone and estradiol	FFQ	Control Depression Vitamin C Quartile 1 Quartile 2 Quartile 3 Quartile 4	Vitamin C (mg/day) 102.0 (66.0, 152.0) 92.0 (56.0, 144.0) 1.00 0.77 (0.60, 0.99) 0.74 (0.56, 0.96) 0.70 (0.52, 0.93)	CES-D	7

### Relative Risk of Depression for the Highest Versus Lowest Dietary Vitamin C Intake Category

**FIGURE 2:**
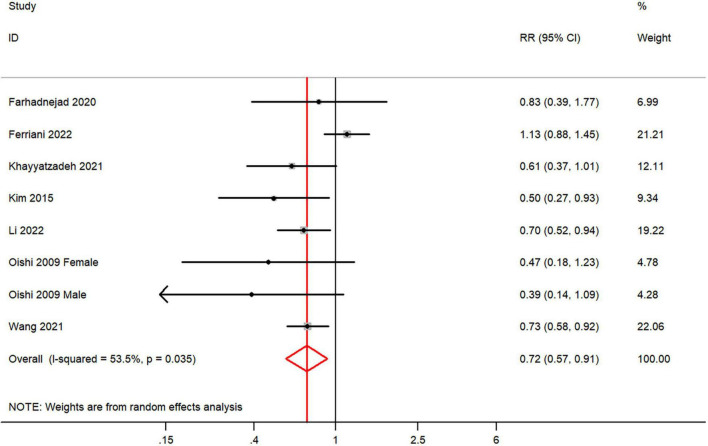
Forest plot of meta-analysis: overall multi-variable adjusted RR of depression for the highest versus lowest category of dietary vitamin C intake.

The overall multi-variable adjusted RR demonstrated that dietary vitamin C intake was negatively associated with depression (RR = 0.72, 95% CI: 0.57 to 0.91; *P* = 0.005) (Figure 2). A substantial level of heterogeneity was observed among various studies (*P* = 0.035, *I*^2^ = 53.5%). No evidence of publication bias existed according to the Begg’s rank-correlation test (*P* = 0.108), and the slope coefficient is 0.036. The results of meta-regression were showed as follow (Supplementary Table 3): publication year (*P* = 0.087), sample size (*P* = 0.296), location (*P* = 0.133), age (*P* = 0.169), sex (*P* = 0.307), dietary assessment (*P* = 0.911). The results of subgroup analysis were presented in Table 2. The negative relationship between dietary vitamin C intake and depression only existed in Asia (RR = 0.57, 95% CI: 0.42 to 0.78; *P* < 0.001), females (RR = 0.69, 95% CI: 0.59 to 0.80; *P* < 0.001), adolescent (RR = 0.61, 95% CI: 0.43 to 0.86; *P* = 0.005) and CES-D or BDI (RR = 0.62, 95% CI: 0.50 to 0.78; *P* < 0.001), but not in non-Asia (RR = 0.84, 95% CI: 0.62 to 1.13; *P* = 0.25), males (RR = 0.89, 95% CI: 0.54 to 1.47; *P* = 0.66), middle aged and elderly (RR = 0.76, 95% CI: 0.58 to 1.02; *P* = 0.07) and other diagnostic criteria of depression (RR = 0.89, 95% CI: 0.63 to 1.26; *P* = 0.52).

**TABLE 2:** Subgroup analysis of depression for the highest versus lowest dietary vitamin C intake category.

Stratification	Number of studies	Pooled RR	95% CI	*P*-value	Heterogeneity
All studies	7	0.72	0.57, 0.91	*P* = 0.005	*P* = 0.04; *I*^2^ = 54%
**Geographical region**					
Asia	4	0.57	0.42, 0.78	*P* < 0.001	*P* = 0.76; *I*^2^ = 0%
Non-Asia	3	0.84	0.62, 1.13	*P* = 0.25	*P* = 0.02; *I*^2^ = 76%
**Dietary assessment**					
FFQ	6	0.70	0.52, 0.94	*P* = 0.02	*P* = 0.02; *I*^2^ = 59%
Recall method	1	0.73	0.58, 0.94	/	/
**Sex**					
Male	3	0.89	0.54, 1.47	*P* = 0.66	*P* = 0.10; *I*^2^ = 57%
Female	7	0.69	0.59, 0.80	*P* < 0.001	*P* = 0.73; *I*^2^ = 0%
**Population**					
Adolescent	3	0.61	0.43, 0.86	*P* = 0.005	*P* = 0.60; *I*^2^ = 0%
Middle aged and elderly	4	0.76	0.58, 1.02	*P* = 0.07	*P* = 0.02; *I*^2^ = 66%
**Diagnostic criteria of depression**					
CES-D or BDI	4	0.62	0.50, 0.78	*P* < 0.001	*P* = 0.70; *I*^2^ = 0%
Others	3	0.89	0.63, 1.26	*P* = 0.52	*P* = 0.04; *I*^2^ = 69%

### Weighted Mean Difference of Dietary Vitamin C Intake for Depression Versus Control Subjects

**FIGURE 3:**
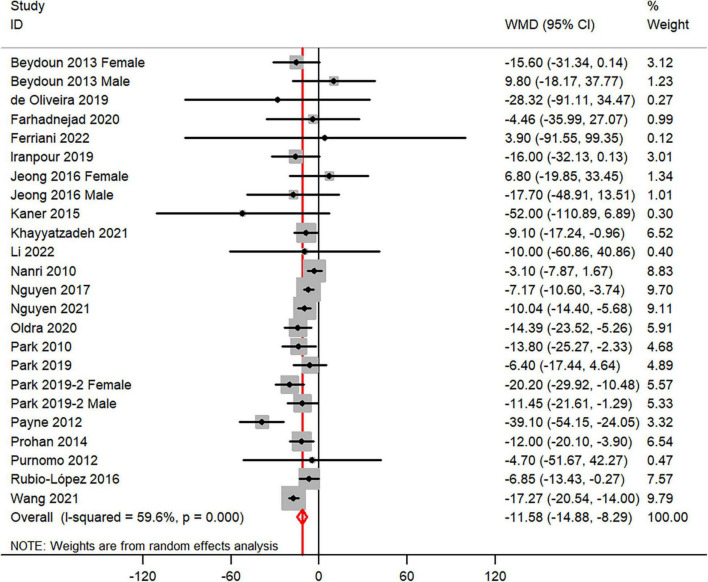
Forest plot of meta-analysis: WMD of dietary vitamin C intake for depression versus control subjects.

The overall combined WMD showed that dietary vitamin C intake in depression was lower than that in control subjects (WMD = −11.58, 95% CI: −14.88 to −8.29; *P* < 0.001) (Figure 3). A substantial level of heterogeneity was observed among the various studies (*P* < 0.001, *I*^2^ = 59.6%). No evidence of publication bias existed according to the Begg’s rank-correlation test (*P* = 0.503), and the slope coefficient is −10.377. The results of meta-regression were showed as follow (Supplementary Table 4): publication year (*P* = 0.661), sample size (*P* = 0.344), location (*P* = 0.068), age (*P* = 0.372), sex (*P* = 0.708), dietary assessment (*P* = 0.358). The results of subgroup analysis were presented in Table 3.

**TABLE 3:** Subgroup analysis for WMD of dietary vitamin C level in depression versus control subjects.

Stratification	Number of studies	Pooled WMD	95% CI	*P*-value	Heterogeneity
All studies	21	−11.58	−14.88, −8.29	*P* < 0.001	*P* < 0.001; *I*^2^ = 60%
**Geographical region**					
Asia	10	−8.31	−10.30, −6.32	*P* < 0.001	*P* = 0.17; *I*^2^ = 28%
Non-Asia	11	−15.61	−21.80, −9.42	*P* < 0.001	*P* = 0.02; *I*^2^ = 51%
**Dietary assessment**					
FFQ	9	−9.70	−16.15, −3.25	*P* = 0.003	*P* = 0.009; *I*^2^ = 59%
Recall method	11	−13.62	−15.71, −11.52	*P* < 0.001	*P* = 0.07; *I*^2^ = 39%
**Sex**					
Male	6	−6.02	−9.97, −2.06	*P* = 0.003	*P* = 0.23; *I*^2^ = 27%
Female	12	−11.74	−14.74, −8.74	*P* < 0.001	*P* = 0.77; *I*^2^ = 0%
**Population**					
Adolescent	6	−9.11	−12.89, −5.34	*P* < 0.001	*P* = 0.86; *I*^2^ = 0%
Middle aged and elderly	15	−12.82	−17.27, −8.37	*P* < 0.001	*P* < 0.001; *I*^2^ = 68%
**Diagnostic criteria of depression**					
CES-D or BDI	11	−7.51	−10.30, −4.72	*P* < 0.001	*P* = 0.53; *I*^2^ = 0%
Others	10	−14.51	−19.61, −9.41	*P* < 0.001	*P* < 0.001; *I*^2^ = 71%

### Relative Risk of Depression for the Highest Versus Lowest Dietary Vitamin E Intake Category

**FIGURE 4:**
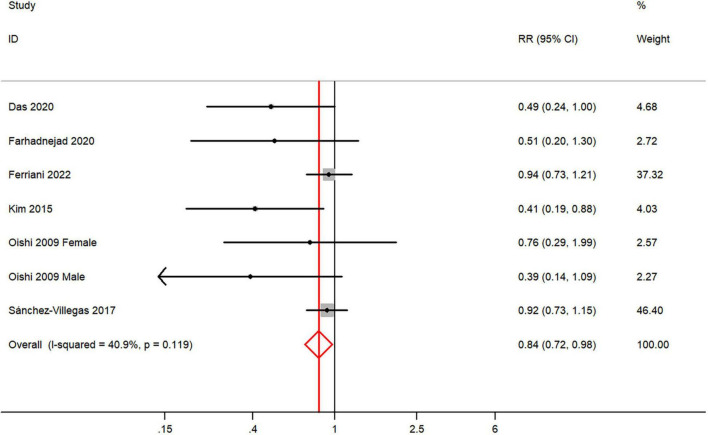
Forest plot of meta-analysis: overall multi-variable adjusted RR of depression for the highest versus lowest category of dietary vitamin E intake.

The overall multi-variable adjusted RR demonstrated that dietary vitamin E intake was negatively associated with depression (RR = 0.84, 95% CI: 0.72 to 0.98; *P* = 0.023) (Figure 4). No substantial level of heterogeneity was observed among various studies (*P* = 0.119, *I*^2^ = 40.9%). No evidence of publication bias existed according to the Begg’s rank-correlation test (*P* = 0.548), and the slope coefficient is 0.150. The results of meta-regression were showed as follow (Supplementary Table 5): publication year (*P* = 0.401), sample size (*P* = 0.031), location (*P* = 0.058), age (*P* = 0.100), sex (*P* = 0.105). The results of subgroup analysis were presented in Table 4. The negative relationship between dietary vitamin E intake and depression only existed in cross-sectional (RR = 0.81, 95% CI: 0.65 to 1.00; *P* = 0.05), Asia (RR = 0.49, 95% CI: 0.31 to 0.77; *P* = 0.002), adolescent (RR = 0.45, 95% CI: 0.25 to 0.81; *P* = 0.008), and CES-D or BDI (RR = 0.48, 95% CI: 0.29 to 0.81; *P* = 0.006), but not in prospective cohort (RR = 0.74, 95% CI: 0.41 to 1.32; *P* = 0.31), non-Asia (RR = 0.90, 95% CI: 0.76 to 1.06; *P* = 0.01), middle aged and elderly (RR = 0.87, 95% CI: 0.75 to 1.03; *P* = 0.10) and other diagnostic criteria of depression (RR = 0.88, 95% CI: 0.75 to 1.04; *P* = 0.13).

**TABLE 4:** Subgroup analysis of depression for the highest versus lowest dietary vitamin E intake category.

Stratification	Number of studies	Pooled RR	95% CI	*P*-value	Heterogeneity
All studies Study design	6	0.84	0.72, 0.98	*P* = 0.02	*P* = 0.12; *I*^2^ = 41%
Cross-sectional	4	0.81	0.65, 1.00	*P* = 0.05	*P* = 0.12; *I*^2^ = 45%
Cohort	2	0.74	0.41, 1.32	*P* = 0.31	*P* = 0.10; *I*^2^ = 4563%
**Geographical region**					
Asia	3	0.49	0.31, 0.77	*P* = 0.002	*P* = 0.75; *I*^2^ = 0%
Non-Asia	3	0.90	0.76, 1.06	*P* = 0.01	*P* = 0.23; *I*^2^ = 32%
**Dietary assessment**					
FFQ	6	0.84	0.72, 0.98	*P* = 0.02	*P* = 0.12; *I*^2^ = 41%
Recall method	/	/	/	/	/
**Sex**					
Male	3	0.61	0.42, 0.90	*P* = 0.19	*P* = 0.39; *I*^2^ = 0%
Female	4	0.73	0.57, 0.94	*P* = 0.02	*P* = 0.34; *I*^2^ = 10%
**Population**					
Adolescent	2	0.45	0.25, 0.81	*P* = 0.008	*P* = 0.72; *I*^2^ = 0%
Middle aged and elderly	4	0.87	0.75, 1.03	*P* = 0.10	*P* = 0.24; *I*^2^ = 27%
**Diagnostic criteria of depression**					
CES-D or BDI	2	0.48	0.29, 0.81	*P* = 0.006	*P* = 0.55; *I*^2^ = 0%
Others	4	0.88	0.75, 1.04	*P* = 0.13	*P* = 0.23; *I*^2^ = 30%

### Weighted Mean Difference of Dietary Vitamin E Intake for Depression Versus Control Subjects

**FIGURE 5:**
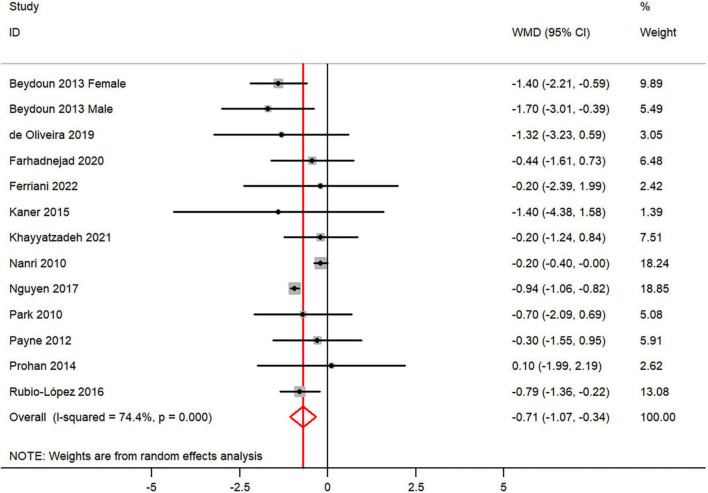
Forest plot of meta-analysis: WMD of dietary vitamin E intake for depression versus control subjects.

The overall combined WMD showed that dietary vitamin E intake in depression was lower than that in control subjects (WMD = −0.71, 95% CI: −1.07 to −0.34; *P* = 0.006) (Figure 5). A substantial level of heterogeneity was observed among the various studies (*P* < 0.001, *I*^2^ = 74.4%). No evidence of publication bias existed according to the Begg’s rank-correlation test (*P* = 0.951), and the slope coefficient is −0.760. The results of meta-regression were showed as follow (Supplementary Table 6): publication year (*P* = 0.737), sample size (*P* = 0.890), location (*P* = 0.164), age (*P* = 0.482), sex (*P* = 0.479), dietary assessment (*P* = 0.083). The results of subgroup analysis were presented in Table 5. The negative relationship between dietary vitamin E intake and depression only existed in recall method (WMD = −1.06, 95% CI: −1.46 to −0.65; *P* < 0.001), female (WMD = −0.40, 95% CI: −0.56 to −0.24; *P* < 0.001), but not in FFQ (WMD = −0.46, 95% CI: −0.98 to 0.06; *P* = 0.08) and males (WMD = −0.41, 95% CI: −1.23 to 0.40; *P* = 0.32).

**TABLE 5:** Subgroup analysis for WMD of dietary vitamin E level in depression versus control subjects.

Stratification	Number of studies	Pooled WMD	95% CI	*P*-value	Heterogeneity
All studies	12	−0.71	−1.07, −0.34	*P* < 0.001	*P* < 0.001; *I*^2^ = 74%
**Geographical region**					
Asia	7	−0.51	−1.02, 0.00	*P* = 0.05	*P* < 0.001; *I*^2^ = 85%
Non-Asia	5	−0.98	−1.37, −0.58	*P* < 0.001	*P* = 0.50; *I*^2^ = 0%
**Dietary assessment**					
FFQ	6	−0.46	−0.98, 0.06	*P* = 0.08	*P* < 0.001; *I*^2^ = 88%
Recall method	5	−1.06	−1.46, −0.65	*P* < 0.001	*P* = 0.73; *I*^2^ = 0%
**Sex**					
Male	4	−0.41	−1.23, 0.40	*P* = 0.32	*P* = 0.11; *I*^2^ = 51%
Female	7	−0.40	−0.56, −0.24	*P* < 0.001	*P* = 0.28; *I*^2^ = 19%
**Population**					
Adolescent	5	−0.60	−1.02, −0.17	*P* = 0.006	*P* = 0.82; *I*^2^ = 0%
Middle aged and elderly	7	−0.83	−1.32, −0.33	*P* = 0.001	*P* < 0.001; *I*^2^ = 84%
**Diagnostic criteria of depression**					
CES-D or BDI	6	−0.28	−0.46, −0.09	*P* = 0.003	*P* = 0.38; *I*^2^ = 6%
Others	6	−0.94	−1.06, −0.82	*P* < 0.001	*P* = 0.57; *I*^2^ = 0%

### Sensitivity Analysis

The results of the sensitivity analysis showed only minimal changes in magnitude of the pooled effect estimate and heterogeneity when any one study was excluded from the meta-analysis, indicating that no individual study had excessive influence on these robust aggregated results (Supplementary Tables 7–10).

## Discussion

A total of 25 observational studies were identified for examination in this meta-analysis, and the pooled analysis showed that both dietary vitamin C and E intake was inversely associated with depression.

The potential beneficial effect of dietary vitamin C and E intake on depression has been demonstrated by experimental evidence. Dulabi et al. finds that the chronic social isolation stress-induced weight gain and depressive-like behavior is protected by vitamin C (47). Moreover, Fraga et al. demonstrates that depressive-like behavior and hippocampal synaptic dysfunction induced by corticosterone is rapidly reversed by a single administration of vitamin C (48). In addition, Koizumi further indicates that vitamin C may impact social environment-related anxiety behavior and stress-induced anorexia in SMP30/GNL knockout mice (49). With regard to vitamin E, Manosso et al. reports that the depressive-like effect induced by TNF-α can be reduced by acute administration of α-tocopherol (30–100 mg/kg) (50). Parveen et al. further shows that the depression-like symptoms can be significantly improved by supplementation of 0.3 ml vitamin E/day for 4 weeks (51). Taken together, these above fundamental evidence strongly supports our results.

Interestingly, some of our findings are only confirmed in females (RR for vitamin C and WMD for vitamin E), which may be explained as follow: (1) the number of study for males is relative limited, which may influence the reliability of subgroup analysis; (2) females may be more precise and reliable in completing the dietary assessment (52); (3) some potential genetic sexual differences in diet-related pathology of depression may exist (53, 54). It should also be noted that some results of RR and WMD were not completely consistent (39). The reasons can be listed as follows: (1) the effect estimate for RR is adjusted by multi-variable, whereas WMD is not; (2) the classification of exposure vary among individuals (tertile or quartile). Therefore, it might be acceptable for the minor difference between RR and WMD result. Importantly, the inverse relationship between dietary vitamin E intake and depression is lost in prospective cohort study. Although the number of prospective cohort studies is rather limited (only 2), the factors that matter the dietary vitamin E intake may change after depression, which may reverse the causality directly (depressive subjects may consume less dietary vitamin E due to the reduced appetite). Moreover, subgroup analysis also suggests that geographic region, dietary assessment, diagnostic criteria of depression and population may influence the overall result. Therefore, these factors may be considered to contribute to the heterogeneity of our study. Taken together, more well-designed prospective cohort studies with sexual specification are still needed.

It should also be noted that vitamin C is a biomarker for vegetable and fruit, which contain a wide range of bioactive constituents for human health: nitrate (55), phytochemicals (56) and folate (57). Phytomedicine may be an alternative and effective treatment for depression when conventional drug is not applicable for its side effects, low effectiveness or inaccessibility (58). Moreover, the serum and dietary level of folate in depression is lower than that in controls (59), and folate supplementation improves the efficacy of traditional antidepressant medications (59). Therefore, folate is considered to be beneficial for the long-term management of depression (60). Taken together, the potential beneficial effect of these above bio-active constituents cannot be fully excluded, which should be considered in further study.

Our study has several strengthens. First, this is the first meta-analysis of observational studies on the associations of dietary vitamin C and E intake with depression. Second, our findings are consistence with the corresponding experimental fundamental evidence, which may provide helpful information to better consider the dietary effect on depression (e.g., vitamin C and E-rich food). The limitations of this study should also be acknowledged. First, the substantial level of heterogeneity might have distorted the reliability of our results. Second, due to the limitation in the relevant literature, only 2 prospective cohort studies are identified (preclude causal relationships). Third, the classification of exposure and diagnostic criteria of depression vary greatly among individuals. Fourth, the selection of adjusted factors is not uniform. Fifth, no included study has considered the severity of depression (e.g., major and minor depression) and dietary quality (the sources of vitamin C and E are partially similar), and some issues cannot be addressed. Last but not the least, the circulating level of vitamin C and E is not considered. These limitations may weaken the significance of this study.

## Conclusion

Our results suggest that both dietary vitamin C and E intake is inversely associated with depression. However, due to the limited evidence, more well-designed prospective cohort studies with sexual specification are still needed.

## Data Availability Statement

The original contributions presented in the study are included in the article/Supplementary Material, further inquiries can be directed to the corresponding author/s.

## Author Contributions

YZ and JD conceived the idea, drafted this manuscript, selected and retrieved relevant manuscript, and assessed each study. JD performed the statistical analysis. YZ was the guarantor of the overall content. Both authors revised and approved the final manuscript.

## Conflict of Interest

The authors declare that the research was conducted in the absence of any commercial or financial relationships that could be construed as a potential conflict of interest.

## Publisher’s Note

All claims expressed in this article are solely those of the authors and do not necessarily represent those of their affiliated organizations, or those of the publisher, the editors and the reviewers. Any product that may be evaluated in this article, or claim that may be made by its manufacturer, is not guaranteed or endorsed by the publisher.

## Supplementary Material

Supplementary Figure 1 Funnel plot with pseudo 95% confidence limits for the analysis of dietary vitamin C intake and depression.

Supplementary Figure 2 Funnel plot with pseudo 95% confidence limits for the dietary vitamin C intake for depression versus control subjects.

Supplementary Figure 3 Funnel plot with pseudo 95% confidence limits for the analysis of dietary vitamin E intake and depression.

Supplementary Figure 4 Funnel plot with pseudo 95% confidence limits for the dietary vitamin E intake for depression versus control subjects.
