# Supplementary figures and images for: Associations of Dietary Vitamin C and E Intake With Depression. A Meta-Analysis of Observational Studies
Source: Front Nutr. 2022 Apr 7;9:857823. doi: 10.3389/fnut.2022.857823 (PMC9021894; doi:10.3389/fnut.2022.857823)

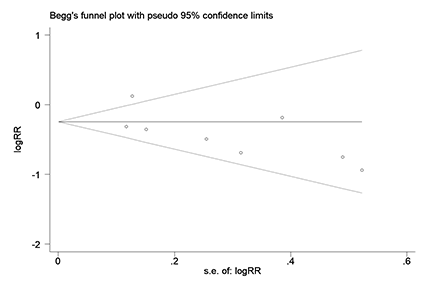

Supplement: Supplementary Figure 1 — Funnel plot with pseudo 95% confidence limits for the analysis of dietary vitamin C intake and depression. [file Image_1.TIF]

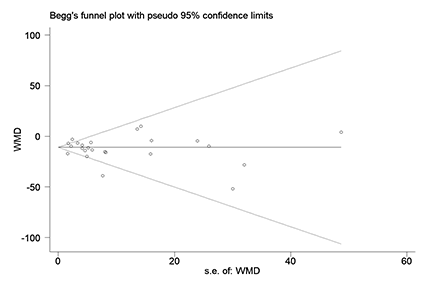

Supplement: Supplementary Figure 2 — Funnel plot with pseudo 95% confidence limits for the dietary vitamin C intake for depression versus control subjects. [file Image_2.TIF]

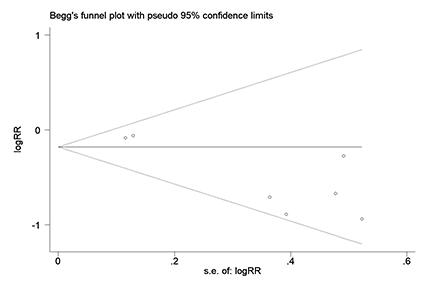

Supplement: Supplementary Figure 3 — Funnel plot with pseudo 95% confidence limits for the analysis of dietary vitamin E intake and depression. [file Image_3.TIF]

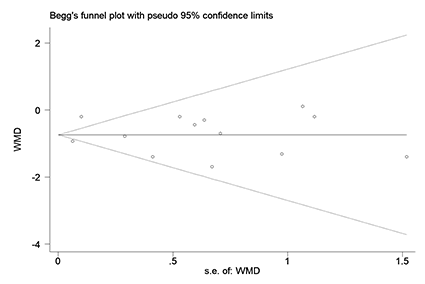

Supplement: Supplementary Figure 4 — Funnel plot with pseudo 95% confidence limits for the dietary vitamin E intake for depression versus control subjects. [file Image_4.TIF]
