# Supplementary material for: Associations of Dietary Vitamin C and E Intake With Depression. A Meta-Analysis of Observational Studies
Source: Front Nutr. 2022 Apr 7;9:857823. doi: 10.3389/fnut.2022.857823 (PMC9021894; doi:10.3389/fnut.2022.857823)
Supplement: Supplementary file 6 [file Table_1.DOCX]

Supplementary table 1 The methodological quality of cross-sectional/case-control studies in accordance with the Newcastle-Ottawa Scale (NOS)

| Study | Selection | | | | Comparability | Exposure | | | Total score |
| --- | --- | --- | --- | --- | --- | --- | --- | --- | --- |
|  | Is the case definition adequate | Representativeness of the cases | Selection of Controls | Definition of Controls | Comparability of cases and controls on the basis of the design or analysis | Ascertainment of exposure | Same method of ascertainment for cases and controls | Non-Response rate |  |
| Oishi 2009 [19] | 1 | 1 | 1 | 1 | 2 | 1 | 1 | 0 | 8 |
| Nanri 2010 [20] | 1 | 1 | 1 | 1 | 1 | 1 | 1 | 0 | 7 |
| Park 2010 [21] | 1 | 0 | 1 | 1 | 2 | 1 | 1 | 0 | 7 |
| Payne 2012 [22] | 1 | 1 | 1 | 1 | 1 | 1 | 1 | 0 | 7 |
| Purnomo 2012 [23] | 1 | 0 | 0 | 1 | 1 | 1 | 1 | 0 | 5 |
| Beydoun 2013 [24] | 1 | 1 | 1 | 1 | 2 | 1 | 1 | 0 | 8 |
| Prohan 2014 [25] | 1 | 0 | 1 | 1 | 1 | 1 | 1 | 0 | 6 |
| Kim 2015 [26] | 1 | 0 | 1 | 1 | 2 | 1 | 1 | 0 | 7 |
| Kaner 2015 [27] | 1 | 0 | 1 | 1 | 1 | 1 | 1 | 0 | 6 |
| Jeong 2016 [28] | 1 | 1 | 1 | 1 | 1 | 1 | 1 | 0 | 7 |
| Rubio-López 2016 [29] | 1 | 0 | 1 | 1 | 2 | 1 | 1 | 0 | 7 |
| Nguyen 2017 [31] | 1 | 1 | 1 | 1 | 1 | 1 | 1 | 0 | 7 |
| de Oliveira 2019 [32] | 1 | 0 | 0 | 1 | 1 | 1 | 1 | 0 | 5 |
| Iranpour 2019 [33] | 1 | 1 | 1 | 1 | 2 | 1 | 1 | 0 | 8 |
| Park 2019 [34] | 1 | 0 | 1 | 1 | 2 | 1 | 1 | 0 | 7 |
| Park 2019-2 [35] | 1 | 1 | 1 | 1 | 1 | 1 | 1 | 0 | 7 |
| Farhadnejad 2020 [37] | 1 | 0 | 1 | 1 | 2 | 1 | 1 | 0 | 7 |
| Oldra 2020 [38] | 1 | 0 | 1 | 1 | 2 | 1 | 1 | 0 | 7 |
| Khayyatzadeh 2021 [39] | 1 | 1 | 1 | 1 | 1 | 1 | 1 | 0 | 7 |
| Wang 2021 [40] | 1 | 1 | 1 | 1 | 2 | 1 | 1 | 0 | 8 |
| Nguyen 2021 [41] | 1 | 1 | 1 | 1 | 2 | 1 | 1 | 0 | 8 |
| Ferriani 2022 [42] | 1 | 1 | 1 | 1 | 2 | 1 | 1 | 1 | 9 |
| Li 2022 [43] | 1 | 0 | 1 | 1 | 2 | 1 | 1 | 0 | 7 |

Supplementary table 2 The methodological quality of cohort studies in accordance with the Newcastle-Ottawa Scale (NOS)

| Study | Selection | | | | Comparability | Outcome | | | Total score |
| --- | --- | --- | --- | --- | --- | --- | --- | --- | --- |
|  | Representativeness of the exposed cohort | Selection of the non-exposed cohort | Ascertainment of exposure | Demonstration that outcome of interest was not present at start of study | Comparability of cohorts on the basis of the design or analysis | Assessment of outcome | Was follow-up long enough for outcomes to occur? | Adequacy of follow up of cohorts? |  |
| Villegas 2017 [30] | 1 | 1 | 1 | 1 | 2 | 1 | 1 | 1 | 9 |
| Das 2020 [36] | 0 | 1 | 1 | 1 | 1 | 1 | 1 | 1 | 7 |

Supplementary Table 3 Results for the meta-regression models for the analysis of dietary vitamin C intake and depression

| Covariate | Coefficient | 95% CI (min) | 95% CI (max) | S.E. | *p-*value |
| --- | --- | --- | --- | --- | --- |
| Publication year | 0.0575 | -0.0114 | 0.1264 | 0.0282 | 0.087 |
| Location | -0.3897 | -0.9387 | 0.1592 | 0.2243 | 0.133 |
| Sample size | 0.00001 | -0.00002 | 0.00004 | 0.00001 | 0.296 |
| Age | -0.3195 | -0.8194 | 0.1804 | 0.2043 | 0.169 |
| Sex | 0.6445 | -0.7694 | 2.0584 | 0.5778 | 0.307 |
| Dietary assessment | -0.0392 | -0.8578 | 0.7795 | 0.3344 | 0.911 |

Supplementary Table 4 Results for the meta-regression models for the dietary vitamin C intake for depression versus control subjects

| Covariate | Coefficient | 95% CI (min) | 95% CI (max) | S.E. | *p-*value |
| --- | --- | --- | --- | --- | --- |
| Publication year | -0.1969 | -1.1151 | 0.7212 | 0.4427 | 0.661 |
| Location | 6.0326 | -0.4894 | 12.5547 | 3.1448 | 0.068 |
| Sample size | -0.00019 | -0.00060 | 0.00022 | 0.0002 | 0.344 |
| Age | -3.3552 | -10.9965 | 4.2862 | 3.6846 | 0.372 |
| Sex | -2.3110 | -14.9347 | 10.3128 | 6.0871 | 0.708 |
| Dietary assessment | 3.1381 | -3.7910 | 10.0671 | 3.3411 | 0.358 |

Supplementary Table 5 Results for the meta-regression models for the analysis of dietary vitamin E intake and depression

| Covariate | Coefficient | 95% CI (min) | 95% CI (max) | S.E. | *p-*value |
| --- | --- | --- | --- | --- | --- |
| Publication year | 0.0330 | -0.0594 | 0.1253 | 0.0359 | 0.401 |
| Location | -0.6040 | -1.2367 | 0.0286 | 0.2461 | 0.058 |
| Sample size | 0.000046 | 0.00001 | 0.00009 | 0.00002 | 0.031 |
| Age | 0.6694 | -0.1850 | 1.5239 | 0.3324 | 0.100 |
| Sex | 0.6540 | -0.1957 | 1.5037 | 0.3306 | 0.105 |

Supplementary Table 6 Results for the meta-regression models for the dietary vitamin E intake for depression versus control subjects

| Covariate | Coefficient | 95% CI (min) | 95% CI (max) | S.E. | *p-*value |
| --- | --- | --- | --- | --- | --- |
| Publication year | -0.0154 | -0.1137 | 0.0830 | 0.0447 | 0.737 |
| Location | 0.4954 | -0.2353 | 1.2262 | 0.3320 | 0.164 |
| Sample size | 0.00002 | -0.00017 | 0.00020 | 0.00008 | 0.890 |
| Age | -0.2682 | -1.0800 | 0.5436 | 0.3688 | 0.482 |
| Sex | 0.4733 | -0.9492 | 1.8958 | 0.6463 | 0.479 |
| Dietary assessment | 0.6392 | -0.0986 | 1.3771 | 0.3353 | 0.083 |

Supplementary Table 7 Results for the sensitivity analysis of dietary vitamin C intake and depression

| Study | Pooled RR | 95% CI | P value | Heterogeneity |
| --- | --- | --- | --- | --- |
| Farhadnejad 2020 | 0.71 | 0.55, 0.91 | P = 0.007 | P = 0.02; I² = 60% |
| Ferriani 2022 | 0.68 | 0.58, 0.79 | P < 0.001 | P = 0.74; I² = 0% |
| Khayyatzadeh 2021 | 0.73 | 0.57, 0.95 | P = 0.02 | P = 0.03; I² = 57% |
| Kim 2015 | 0.75 | 0.59, 0.95 | P = 0.02 | P = 0.04; I² = 54% |
| Li 2022 | 0.71 | 0.54, 0.95 | P = 0.02 | P = 0.03; I² = 58% |
| Oishi 2009 | 0.76 | 0.60, 0.96 | P = 0.02 | P = 0.03; I² = 59% |
| Wang 2021 | 0.70 | 0.52, 0.94 | P = 0.02 | P = 0.02; I² = 59% |

Supplementary Table 8 Results for the sensitivity analysis of dietary vitamin C intake for depression versus control subjects

| Study | Pooled WMD | 95% CI | P value | Heterogeneity |
| --- | --- | --- | --- | --- |
| Beydoun 2013 | -11.73 | -15.10, -8.36 | P < 0.001 | P < 0.001; I² = 61% |
| de Oliveira 2019 | -11.55 | -14.86, -8.23 | P < 0.001 | P < 0.001; I² = 61% |
| Farhadnejad 2020 | -11.67 | -15.00, -8.33 | P < 0.001 | P < 0.001; I² = 61% |
| Ferriani 2022 | -11.61 | -14.93, -8.29 | P < 0.001 | P < 0.001; I² = 61% |
| Iranpour 2019 | -11.46 | -14.83, -8.08 | P < 0.001 | P < 0.001; I² = 61% |
| Jeong 2016 | -11.78 | -15.12, -8.43 | P < 0.001 | P < 0.001; I² = 62% |
| Kaner 2015 | -11.45 | -14.72, -8.18 | P < 0.001 | P < 0.001; I² = 60% |
| Khayyatzadeh 2021 | -11.79 | -15.27, -8.30 | P < 0.001 | P < 0.001; I² = 61% |
| Li 2022 | -11.60 | -14.93, -8.27 | P < 0.001 | P < 0.001; I² = 61% |
| Nanri 2010 | -12.35 | -15.56, -9.13 | P < 0.001 | P = 0.003; I² = 51% |
| Nguyen 2017 | -12.08 | -15.65, -8.52 | P < 0.001 | P < 0.001; I² = 57% |
| Nguyen 2021 | -11.81 | -15.47, -8.15 | P < 0.001 | P < 0.001; I² = 61% |
| Oldra 2020 | -11.43 | -14.88, -7.98 | P < 0.001 | P < 0.001; I² = 61% |
| Park 2010 | -11.49 | -14.92, -8.07 | P < 0.001 | P < 0.001; I² = 61% |
| Park 2019 | -11.87 | -15.28, -8.45 | P < 0.001 | P < 0.001; I² = 61% |
| Park 2019-2 | -11.06 | -14.57, -7.55 | P < 0.001 | P < 0.001; I² = 61% |
| Payne 2012 | -10.69 | -12.29, -9.09 | P < 0.001 | P = 0.004; I² = 49% |
| Prohan 2014 | -11.59 | -15.08, -8.10 | P < 0.001 | P < 0.001; I² = 61% |
| Purnomo 2012 | -11.63 | -14.96, -8.30 | P < 0.001 | P < 0.001; I² = 61% |
| Rubio-López 2016 | -11.99 | -15.48, -8.51 | P < 0.001 | P < 0.001; I² = 60% |
| Wang 2021 | -9.06 | -10.88, -7.24 | P < 0.001 | P = 0.02; I² = 43% |

Supplementary Table 9 Results for the sensitivity analysis of dietary vitamin E intake and depression

| Study | Pooled RR | 95% CI | P value | Heterogeneity |
| --- | --- | --- | --- | --- |
| Das 2020 | 0.86 | 0.73, 1.01 | P = 0.06 | P = 0.16; I² = 37% |
| Farhadnejad 2020 | 0.85 | 0.72, 0.99 | P = 0.04 | P = 0.11; I² = 45% |
| Ferriani 2022 | 0.78 | 0.64, 0.95 | P = 0.01 | P = 0.12; I² = 43% |
| Kim 2015 | 0.86 | 0.74, 1.01 | P = 0.06 | P = 0.24; I² = 26% |
| Oishi 2009 | 0.85 | 0.73, 1.00 | P = 0.05 | P = 0.09; I² = 49% |
| Sánchez-Villegas 2017 | 0.77 | 0.62, 0.95 | P = 0.02 | P = 0.11; I² = 44% |

Supplementary Table 10 Results for the sensitivity analysis of dietary vitamin E intake for depression versus control subjects

| Study | Pooled WMD | 95% CI | P value | Heterogeneity |
| --- | --- | --- | --- | --- |
| Beydoun 2013 | -0.56 | -0.96, -0.17 | P = 0.005 | P < 0.001; I² = 76% |
| de Oliveira 2019 | -0.69 | -1.06, -0.32 | P < 0.001 | P < 0.001; I² = 76% |
| Farhadnejad 2020 | -0.73 | -1.11, -0.35 | P < 0.001 | P < 0.001; I² = 76% |
| Ferriani 2022 | -0.72 | -1.09, -0.35 | P < 0.001 | P < 0.001; I² = 76% |
| Kaner 2015 | -0.70 | -1.07, -0.33 | P < 0.001 | P < 0.001; I² = 76% |
| Khayyatzadeh 2021 | -0.75 | -1.13, -0.37 | P < 0.001 | P < 0.001; I² = 76% |
| Nanri 2010 | -0.92 | -1.04, -0.81 | P < 0.001 | P = 0.70; I² = 0% |
| Nguyen 2017 | -0.36 | -0.53, -0.19 | P < 0.001 | P = 0.11; I² = 35% |
| Park 2010 | -0.71 | -1.09, -0.33 | P < 0.001 | P < 0.001; I² = 77% |
| Payne 2012 | -0.73 | -1.11, -0.35 | P < 0.001 | P < 0.001; I² = 76% |
| Prohan 2014 | -0.73 | -1.10, -0.36 | P < 0.001 | P < 0.001; I² = 76% |
| Rubio-López 2016 | -0.70 | -1.10, -0.29 | P < 0.001 | P < 0.001; I² = 76% |
